# Effectiveness of TOcilizumab in comparison to Prednisone In Rheumatoid Arthritis patients with insufficient response to disease-modifying antirheumatic drugs (TOPIRA): study protocol for a pragmatic trial

**DOI:** 10.1186/s13063-020-04260-y

**Published:** 2020-04-05

**Authors:** Matthijs S. van der Leeuw, Paco M. J. Welsing, Maria J. H. de Hair, Johannes W. G. Jacobs, Anne C. A. Marijnissen, Suzanne P. Linn-Rasker, Faouzia Fodili, Reinhard Bos, Janneke Tekstra, Jacob M. van Laar

**Affiliations:** 1 University Medical Center Utrecht, Heidelberglaan 100, 3584 CX Utrecht, The Netherlands; 2 Meander Medical Center, Maatweg 3, 3813 TZ Amersfoort, The Netherlands; 3 Reumazorg Zuid West Nederland, Streuvelslaan 18, 4707 CH Roosendaal, The Netherlands; 4 Medical Center Leeuwarden, Henri Dunantweg 2, 8934 AD Leeuwarden, The Netherlands

**Keywords:** Rheumatoid arthritis, Tocilizumab, Prednisone, Randomized controlled trial, Insufficient response to csDMARDs

## Abstract

**Background:**

Rheumatoid arthritis (RA) is a chronic inflammatory autoimmune disease, predominantly affecting joints, which is initially treated with conventional synthetic disease-modifying antirheumatic drugs (csDMARDs). In RA patients with insufficient response to csDMARDs, the addition of prednisone or tocilizumab, a biological DMARD (bDMARD), to the medication has been shown to be effective in reducing RA symptoms. However, which of these two treatment strategies has superior effectiveness and safety is unknown.

**Methods:**

In this multicenter, investigator-initiated, open-label, randomized, pragmatic trial, we aim to recruit 120 RA patients meeting the 2010 ACR/EULAR classification criteria for RA, with active disease defined as a Clinical Disease Activity Index (CDAI) > 10 and at least one swollen joint of the 28 assessed. Patients must be on stable treatment with csDMARDs for ≥ 8 weeks prior to screening and must have been treated with ≥ 2 DMARDs, of which a maximum of one tumor necrosis factor inhibitor (a class of bDMARDs) is allowed. Previous use of other bDMARDs or targeted synthetic DMARDs is not allowed. Patients will be randomized in a 1:1 ratio to receive either tocilizumab (subcutaneously at 162 mg/week) or prednisone (orally at 10 mg/day) as an addition to their current csDMARD therapy. Study visits will be performed at screening; baseline; and months 1, 2, 3, 6, 9, and 12. Study medication will be tapered in case of clinical remission (CDAI ≤ 2.8 and ≤ 1 swollen joint at two consecutive 3-monthly visits) with careful monitoring of disease activity. In case of persistent high disease activity at or after month 3 (CDAI > 22 at any visit or > 10 at two consecutive visits), patients will switch to the other strategy arm. Primary outcome is a change in CDAI from baseline to 12 months. Secondary outcomes are additional clinical response and quality of life measures, drug retention rate, radiographically detectable progression of joint damage, functional ability, and cost utility. Safety outcomes include tocilizumab-associated adverse events (AEs), glucocorticoid-associated AEs, and serious AEs.

**Discussion:**

This will be the first randomized clinical trial comparing addition of oral prednisone or of tocilizumab head to head in RA patients with insufficient response to csDMARD therapy. It will yield important information for clinical rheumatology practice.

**Trial registration:**

This trial was prospectively registered in the Netherlands Trial Register on October 7, 2019 (NL8070). The Netherlands Trial Register contains all items from the World Health Organization Trial Registration Data Set.

## Administrative information

**Table:** 

**Title**	Effectiveness of TOcilizumab in comparison to Prednisone In Rheumatoid Arthritis patients with insufficient response to disease modifying anti-rheumatic drugs (TOPIRA): study protocol for a pragmatic trial.
**Trial registration**	Netherlands Trial Register: NL8070
**Protocol version**	Protocol version 1.2 (September 20, 2019)
**Funding**	Funding is provided by Hoffman-La Roche (no conditions set)
**Author details**	*Department of Rheumatology & Clinical Immunology, University Medical Center Utrecht (UMCU)* M.S. van der Leeuw, P.M.J. Welsing, M.J. de Hair, A.C.A. Marijnissen, J. Tekstra, J.W.G. Jacobs, J.M. van Laar *Rheumatology department of Meander Medisch Centrum, Amersfoort, The Netherlands* S.P. Linn-Rasker *Reumazorg Zuid West Nederland, Roosendaal, The Netherlands* F. Fodili *Rheumatology department of Medisch Centrum Leeuwarden, The Netherlands* R. Bos
**Name and contact information for the trial sponsor**	Sponsor: University Medical Center Utrecht (UMCU), Heidelberglaan 100, 3584 CX, Utrecht, The Netherlands. Postal address: G02.228, P.O. Box 85500, 3508GA, Utrecht, The Netherlands. Contact: M.S. van der Leeuw, m.s.vanderleeuw-17@umcutrecht.nl
**Role of sponsor**	This is an investigator-initiated trial by the UMCU. The UMCU is responsible for the study design; collection, management, analysis, interpretation of data writing of the report; and the decision where to submit the report for publication. The UMCU will have ultimate authority over these activities.

## Background

Rheumatoid arthritis (RA) is a chronic inflammatory autoimmune disease associated with joint damage, chronic pain, fatigue, and functional disability, which can lead to significantly reduced work participation and health-related quality of life [1]. Treatment options for RA comprise several different classes of immunosuppressive medication, called disease-modifying antirheumatic drugs (DMARDs). The first choice is treatment with a conventional synthetic DMARD (csDMARD), like methotrexate (MTX), which has broad immunosuppressive effects and is the cornerstone in the treatment of RA. Often, MTX is temporarily (3–6 months) combined with glucocorticoid (GC) therapy, which has an even broader immunosuppressive effect [2]. GCs are not categorized as DMARDs, but they do have DMARD properties [3]. However, due to the attribution of adverse effects, the long-term use of GCs in RA is still controversial [4, 5]. Over the past 20 years, several new (classes of) DMARDs have been developed, consisting of different types of biological DMARDS (bDMARDs) and more recently targeted synthetic DMARDs (tsDMARDs). These specifically inhibit a single pro-inflammatory pathway (in contrast to the broad effect of csDMARDs) and are usually added to the therapeutic strategy in case of insufficient response to csDMARD therapy [6].

Upon diagnosis of RA, treatment with csDMARDs should be initiated as soon as possible. Patients are treated long-term to reduce signs and symptoms and to reduce or even prevent joint damage and disability. The current primary treatment target for RA is to achieve a state of clinical remission (i.e., no signs or symptoms of RA) or low disease activity, as this has been shown to improve not only the short-term but also the long-term outcome [6]. Patients should be monitored regularly for therapeutic efficacy and adverse effects, and the treatment strategy should be readily adjusted if the treatment target is not met (the principles of “tight control” and “treat-to-target” strategies) [2].

In patients who do not achieve the treatment target with csDMARD therapy, a bDMARD, tsDMARD, or long-term low-dose prednisone can be added to their csDMARD therapy. In current clinical practice, many patients start with the addition of a bDMARD. Several classes of bDMARDs exist, including tumor necrosis factor inhibitors (TNFi), interleukin (IL)-6-receptor blockers, co-stimulatory signal blockers, and anti-B cell therapies. Current guidelines for treatment of RA do not state a preference for a specific class to be added as the first bDMARD, since they are all regarded as approximately equally effective and safe as a group [7]. These bDMARDs are efficacious but also expensive, mostly costing over €10,000/year for one patient [8]. Prednisone is a glucocorticoid that comes at a significantly lower cost (€40/year for 10 mg daily) [9]. The addition of prednisone has been shown to be efficacious [10] but is generally associated with adverse effects when used long-term or in higher dosages. Therefore, it is often regarded as less suitable for the long-term treatment of RA, and currently, treatment in the lowest possible effective dose, with tapering as quickly as clinically possible (within 3–6 months), is being recommended [2]. However, a meta-analysis of multiple randomized trials comparing the long-term (2 years) addition of a low-moderate dose prednisone (5-10 mg/day) to placebo in RA, showed only mild toxicity of prednisone compared to the placebo [11]. Therefore the question arises of which strategy (the addition of prednisone or of a bDMARD) has superior effectiveness, safety, and cost-effectiveness.

No head-to-head studies have been performed comparing the effectiveness of a bDMARD with that of low-moderate dose prednisone. However, one indirect comparison has recently been published that assessed the additive effect of moderate-low dose prednisone versus the bDMARD tocilizumab (TCZ), using data from two trials in csDMARD-naïve early RA patients [12]. The first trial to make this comparison demonstrated that 10 mg of prednisone in addition to MTX in a treat-to-target strategy aiming at remission was more effective in reducing disease activity compared to MTX plus placebo over 2 years of follow-up [5]. The second trial, performed in a similar patient population, showed that a treatment strategy aiming at remission by the initiation of TCZ with MTX was more effective compared with initiation of MTX plus placebo over 2 years of follow-up [13]. The indirect comparison of these two trials used individual patient data and showed lower disease activity for MTX + TCZ compared with MTX + prednisone, as measured using the Disease Activity Score-28 (DAS28). The DAS28 (see Additional file 1) is a composite index of joint swelling and tenderness, a patient’s assessment of global health, and an acute phase reactant (either C-reactive protein or erythrocyte sedimentation rate). However, since TCZ has been shown to have specific inhibitory effects on acute phase reactants independent of clinical disease activity measures such as joint tenderness and swelling, the DAS28 is not suitable for assessing the effects of TCZ [14]. Accordingly, the indirect comparison showed no significant difference on clinical outcome measures alone (using a modified version of the Clinical Disease Activity Index). Also, it is important to note that these trials were performed in DMARD-naïve early RA patients, for which bDMARDs like TCZ are currently not recommended [2]. The extent to which these results will also apply to RA patients who have failed on csDMARDs and for whom the decision to add either prednisone or a bDMARD is relevant is unknown.

### Objectives

A direct comparison of these two treatment strategies on effectiveness, safety, and costs is crucial to be able to make a substantiated choice between adding low-moderate dose prednisone or a bDMARD when csDMARDs have failed. The aim of this study is to assess the clinical effectiveness of treatment with the bDMARD tocilizumab (an IL-6 receptor blocker) compared to treatment with 10 mg prednisone daily, both as addition to current csDMARD therapy in a treat-to-target strategy. Furthermore, we will assess radiographic progression (i.e., the increase of bone erosion and joint space narrowing), safety, and patient-reported outcomes, and will perform a cost-effectiveness analysis.

If the addition of TCZ is more effective than prednisone, TCZ may partly replace the use of long-term glucocorticoids [15, 16]. However, even when TCZ is (slightly) more effective, given the low cost of prednisone compared to TCZ therapy (± €15,000 per year), the addition of prednisone may still be a highly cost-effective approach (price based on list prices in the Netherlands).

## Methods/Design

This multicenter study is an investigator-initiated, pragmatic, randomized, open-label trial coordinated by the Department of Rheumatology & Clinical Immunology of the University Medical Center Utrecht (UMCU).

Study participants will be recruited through the outpatient rheumatology clinics of participating centers. Multiple treatment clinics for rheumatic diseases in the Netherlands will participate, and a current list of participating centers will be updated in the Netherlands Trials Register and EUDRA-CT. Patients will be asked by their treating rheumatologist for their permission to be informed about the study protocol by a research physician. If the patient agrees to participate, the patient will be invited for the screening study visit at which the informed consent form will be signed, and in- and exclusion criteria will be checked. In the informed consent forms, patients are also asked for permission to participate in an ancillary study, in which a HandScan [17] will be performed at every 3-monthly visit, as well permission to use their anonymized data for future research. All study data will be entered in the online electronic Case Report Form (eCRF) Castor Electronic Date Capture by the investigating personnel. This is a secure cloud-based platform that contains automatic range checks, and study IDs are used to pseudonymize all data.

### Population

#### Inclusion criteria

Patients are included if they meet the following criteria:

- are able and willing to give written informed consent
- have sufficient knowledge of the Dutch language to be able to comply with the requirements of the study protocol
- are at least 18 years of age
- are diagnosed as having RA by their rheumatologist and meet the 2010 ACR/EULAR classification criteria for RA [18]
- have active RA defined as a Clinical Disease Activity Index (CDAI) > 10 and at least one swollen joint of the 28 joint count (see Additional file 1)
- are on stable treatment with csDMARD therapy for ≥ 8 weeks prior to the screening visit
- have had previous treatment with ≥2 DMARDs, of which a maximum of one TNF-inhibitor is allowed

#### Exclusion criteria

Patients are excluded according to the following criteria:

- have a contraindication for treatment with oral prednisone as determined by the treating rheumatologist, in line with regular care
- have a contraindication for treatment with TCZ as determined by the treating rheumatologist, or as described in the Summary of Product Characteristics (SPC)
- used systemic GCs (including intra-articular GCs) within 4 weeks before the screening visit
- currently use or previously used a bDMARD or tsDMARD, although previous use of a maximum of one TNF-inhibitor is allowed
- received treatment with any investigational agent within 4 weeks prior to the screening visit
- have any other inflammatory rheumatic disease than RA, except for secondary Sjögren’s syndrome
- are pregnant (assessed with a pregnancy test) or breast feeding or are considering becoming pregnant during the study period

These inclusion and exclusion criteria aim to include RA patients eligible to start on their first bDMARD (i.e., having failed on two previous csDMARDs and not having used any other bDMARDs/tsDMARDs). However, a large part of our patient population will have used a TNFi in the past. To increase the eligible population, we decided to also include patients who have used a maximum of one TNFi. Since most RA patients will have used GCs in the past, it was not feasible to exclude these patients.

### Sample size

A sample size of 50 in each group will have 80% power to detect a difference between groups of eight in the primary endpoint (change in CDAI from baseline to 12 months) using an alpha level of 0.05 and assuming a standard deviation of 14 (as calculated using G*Power 3.1). This difference corresponds to two-thirds of the minimally clinically important difference in CDAI, as presented in a previous study [19], and represents an effect size of approximately 0.57 (Cohen’s *d*), which is considered a moderate effect size. Increasing the sample by 20% for possible dropouts or missing data means we will include 60 patients per group in the study. This will account for a dropout rate of 17% which is similar to dropout rates in previous studies performed by our hospital.

### Study design

Patients who are eligible and have given written informed consent will be randomized in a 1:1 ratio at the screening visit to treatment with either TCZ at 162 mg/week subcutaneously or to prednisone 10 mg/day orally in addition to their current csDMARD therapy. Randomization will be performed by the validated randomization algorithm in the electronic Case Report Form (eCRF) Castor EDC system, stratified by center using random block sizes. To ensure concealment, the block sizes will not be disclosed. The algorithm is unknown to any of the investigators, thereby ensuring allocation concealment. After allocation, treatment is not blinded.

Screening for infectious diseases will be performed in both groups and consists of screening for hepatitis B (HBsAg), hepatitis C (HCV), and tuberculosis (chest X-ray and Quantiferon test). Any positive outcome of the screening will exclude a patient from the study. In addition, a pregnancy test (in either blood or urine) will be performed in all premenopausal women before the start of any study medication, and a positive outcome will exclude the patient.

Patients will be evaluated at screening; baseline; at month 1, 2, and 3, and every 3 months thereafter through the 12-month follow-up. If necessary, the treating rheumatologist can plan an additional unscheduled safety visit, e.g., in case of side-effects or complaints of high disease activity.

### Treatment strategies

TCZ will be supplied in pre-filled syringes of 162 mg, which can be administered subcutaneously by the patient at home. Prednisone will be supplied in tablets of 5 mg. All patients randomized to the prednisone group will additionally receive alendronic acid at 70 mg/week in combination with calcium (1000 mg/day) and vitamin D (800 IU/day), which is standard care for patients receiving long-term prednisone to prevent GC-induced osteoporosis. The calcium dosage may be lowered by the treating rheumatologist based on the patients’ dairy intake. The csDMARD therapy will be continued in a stable dose during the study for both treatment arms. The addition of extra DMARDs (including systemic GCs) is not allowed during the study. However, patients are allowed to receive non-steroidal anti-inflammatory drugs (NSAIDs) and intra-articular GCs during the study, which will be recorded.

Treatment targets are set using the Clinical Disease Activity Index (CDAI, see Additional file 1), which is calculated as the number of tender joints (tender joint count/TJC) + the number of swollen joints (swollen joint count/SJC) + the Visual Analog Scale to assess Global Disease Activity (VAS GDA) as determined by the patient + VAS GDA as determined by the evaluator (both on a scale of 0.0–10.0). The joint counts can each have a maximum score of 28 with the following joints being assessed: the proximal interphalangeal joints of the hands, metacarpophalangeal joints, wrists, elbows, shoulders, and knees. Swelling or tenderness of other joints will not be assessed in this study.

Patients will switch to the treatment used in the other treatment group if any of the following cases arise:

- Moderate disease activity (CDAI > 10) at two consecutive visits, of which the first visit is at or after month 3
- High disease activity (CDAI > 22) at any visit at or after month 3
- If the treating rheumatologist determines it is necessary to stop the study medication because of medical reasons

If, after a switch, the disease remains persistently active (according to the aforementioned criteria) or the treating rheumatologist concludes that the medication needs to be stopped for medical reasons, patients will receive the standard of care (usually another class of bDMARDs or a tsDMARD).

If in a state of clinical remission (CDAI ≤2.8 AND a maximum of one swollen joint of the 28 joint count) at two consecutive 3-monthly study visits (either at 3 and 6 months or at 6 and 9 months), the study medication will be tapered. For TCZ, the dosage interval will be increased from 162 mg every week to 162 mg every 2 weeks. This interval will not be increased further in case of persistent remission. Prednisone will be tapered according to a predefined schedule, in which the dose is gradually reduced with 2.5 mg every 3 months (see Additional file 2). This schedule is in line with local clinical practice. This means that after 12 months, the dose may have been tapered from 10 mg/day to 5 mg/day. If patients experience moderate-high disease activity during tapering (defined as a CDAI > 10), they will be treated with the last effective dose.

In case of adverse events, the dosage of prednisone or administration frequency of TCZ can be altered temporarily (e.g., for TCZ stopped in case of an infection). This decision is left to the treating rheumatologist. When the adverse event has resolved, the medication may be continued if deemed appropriate by the treating rheumatologist. The treating rheumatologist can always deviate from the protocol if this is necessary for medical reasons.

**Fig. 1:**
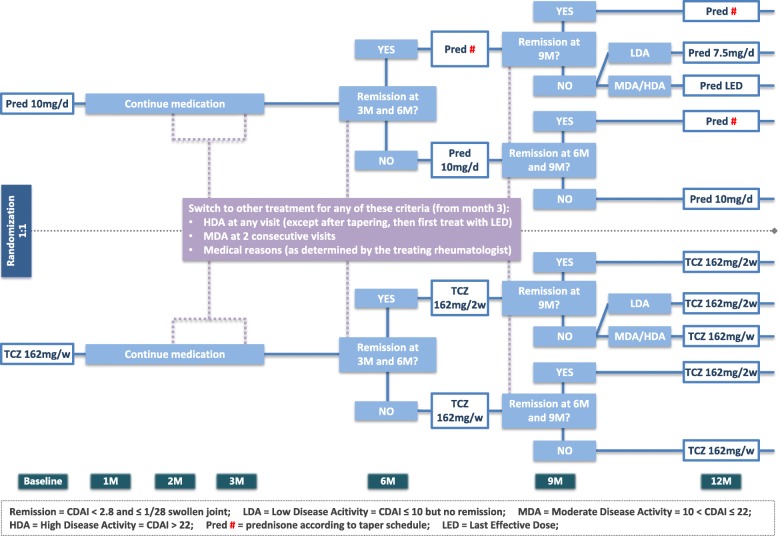
Overview of study design. Remission = CDAI ≤ 2.8 and ≤ 1/28 swollen joints, LDA = low disease activity = CDAI ≤ 10 but no remission, MDA = moderate disease activity = 10 < CDAI ≤ 22, HDA = high disease activity = CDAI > 22, Pred # = prednisone according to taper schedule, LED = last effective dose

See Fig. 1 for an overview of the study design. Additional file 3 contains the Standard Protocol Items: Recommendations for Interventional Trials (SPIRIT) checklist.

### Withdrawal

Subjects can leave the study at any time for any reason if they wish to do so, without any consequences. Patients will be asked if they want to withdraw from the study completely, or if they only want to cease study medication, and complete the follow-up. The treating rheumatologist can decide to withdraw a subject from the study for urgent medical reasons. Patients who withdraw during the study will not be replaced.

### Endpoints

#### Primary endpoint

The primary endpoint will be change in CDAI from baseline to 12 months. The CDAI was chosen as primary endpoint because acute phase reactants, which are strongly and specifically reduced by TCZ independent of clinical RA disease activity, are not a component of this disease activity index (as opposed to the DAS28) [14].

#### Secondary endpoints

##### Clinical outcome measures

Clinical outcome measures include the following:

- Change in Disease Activity Score assessing 28 joints (DAS28) and Simple Disease Activity Index (SDAI) from baseline to 12 months (see Additional file 1).
- ACR20, ACR50, and ACR70 response and EULAR good or moderate response (see Additional file 4).
- Proportion of patients who at any visit reach a state of clinical remission. We will assess four definitions of remission: CDAI ≤ 2.8 AND a maximum of one swollen joint of the 28 joint count; SDAI ≤ 3; DAS28 < 2.6; and ACR-EULAR Boolean remission (defined as a tender joint count ≤ 1, a swollen joint count ≤ 1, a C-reactive protein ≤ 1 mg/dL, and a patient global assessment of disease activity ≤ 1 on a 0–10 scale).
- Radiographic progression from baseline to 12 months (i.e., an increase of bone erosion and joint space narrowing on X-rays of both hand and feet) using a validated score (Sharp-van der Heijde score), determined by an assessor who is blinded for the treatment arm. Scoring of radiographic progression will be done pair-wise and chronologically, which means the assessor scores the X-rays in pairs of the same patient and is aware of the chronological order in which the X-rays were taken (i.e., baseline and 12 months follow-up).
- Drug retention rate and drug compliance (using medication diaries and overviews of drug supply by the pharmacy). Because returning and counting empty syringes of subcutaneous TCZ lead to a risk of needle stick injury, we will not ask the patients to return syringes.

##### Patient-reported outcomes

Questionnaires will be sent by email or distributed on paper at every 3-monthly visit. The following patient-reported outcomes will be assessed:

- Change in pain from baseline to 12 months using a visual analog scale (VAS) ranging from 0 to 10, which assesses the amount of pain experienced on that day.
- Change in quality of life from baseline to 12 months (using the utility score calculated from the EQ-5D-5 L) [20].
- Change in functional ability from baseline to 12 months (using the Dutch consensus Health Assessment Questionnaire/HAQ) [21].
- Change in fatigue (using the FACIT-Fatigue), sleep quality (using the Pittsburg Sleep Quality Index/PSQI) and anxiety and depression (using the Hospital Anxiety and Depression Scale/HADS) from baseline to 12 months [22–24].

##### Safety outcomes

- Laboratory assessment consisting of blood cell count, creatinine, alanine transaminase (ALAT), and glucose will be performed at every visit. HbA1C and low-density lipoprotein (LDL)-cholesterol will be collected at every 3-monthly visit.
- Bone mineral density will be assessed at baseline and 12 months using DEXA scans (Dual Energy X-ray Absorptiometry).
- Serious adverse events (SAEs) and all adverse events (AEs) reported spontaneously by the patient or observed by the investigator or staff will be recorded.
- If the treating rheumatologist has decided the medication needs to be stopped for medical reasons, or if the treatment deviates from the protocol, the reasons for these deviations will be recorded.
- In addition, we will explicitly evaluate in a standardized way the occurrence of AEs associated with GCs, using a recently developed Glucocorticoid Toxicity Index (GTI) [25], and with TCZ, using a predefined list of TCZ-associated AEs, as defined in Table 1. These will both be assessed in each treatment arm to allow for comparison of total AEs.

**Table 1:** Tocilizumab-associated adverse events

Category	Additional criteria
Serious and/or medically significant infections	Opportunistic infections Infections treated with intravenous (IV) anti-infectives
Myocardial infarction (MI)/ acute coronary syndrome (ACS)	All MI/ACS events
Gastrointestinal perforations	Gastrointestinal perforation includes related fistulae and related intra-abdominal abscesses
Malignancies	All malignancies
Anaphylaxis/hypersensitivity reactions	Anaphylaxis as per Sampson’s criteria [26] Hypersensitivity based on investigators’ medical judgment
Demyelinating disorders	All demyelinating disorders
Stroke	Includes stroke and transient ischemic attack (TIA) events
Serious and/or medically significant bleeding events	Bleeding requiring transfusion Bleeding with hospital visit for evaluation (including emergency department for outpatient clinic)
Serious and/or medically significant hepatic events	Event with hepatic clinical diagnosis Meets hepatic laboratory criteria for Hy’s Law^a^ Hepatic laboratory abnormality resulting in TCZ withdrawal (i.e., permanent discontinuation of TCZ therapy)

^a^ ALAT or ASAT concentrations greater than three times the upper limit AND serum bilirubin greater than two times the upper limit (without cholestasis) AND no other reason than the medication to explain these values

### Cost-effectiveness

Observed antirheumatic drug use and visits to the rheumatology outpatient clinic will be recorded. Direct medical (e.g., general practitioner visits) and nonmedical (e.g., travel expenses) costs as well as indirect costs (e.g., productivity loss) will be obtained using a Health Care Utilization and Work Productivity Questionnaire. The incremental cost-effectiveness ratio and incremental cost-utility ratio will be calculated using change in CDAI and quality-adjusted life years (calculated using the EQ-5D-5 L), respectively. The cost-utility analysis will be performed in line with the Dutch Guideline for economic evaluation [27].

**Fig. 2:**
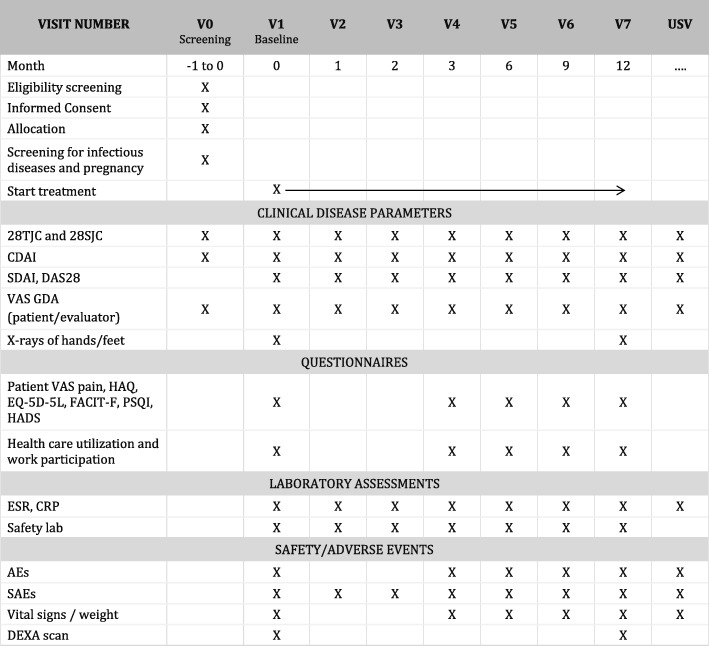
SPIRIT Figure: Overview of study assessments*.* USV: unscheduled safety visit. For patients experiencing side-effects or complaints of high disease activity, an unscheduled safety visit can be planned

Study outcome assessments per visit are summarized in the SPIRIT figure (Fig. 2).

### General characteristics

We will collect the following general patient characteristics at baseline: demographic data (age, sex, and disease duration), smoking status and history, current alcohol use, medical history (using the Charlson Comorbidity index), previous treatment for RA, and status for anti-citrullinated protein antibodies (ACPA) and rheumatoid factor (RF). At every visit we will assess C-reactive protein (CRP) and erythrocyte sedimentation rate (ESR). At every 3-monthly visit we will record weight, blood pressure, and pulse rate. Use of analgesics and current treatment for RA (including intra-articular GCs) will be recorded at every 3-monthly visit.

### Statistical analysis

#### Primary endpoint

The change in CDAI from baseline to 12 months will be compared between the TCZ and prednisone groups using analysis of covariance (ANCOVA), adjusting for the baseline CDAI value, for center, and for the previous use of a TNF-inhibitor. In case the data is not normally distributed, a transformation will be used to normalize data as appropriate. This analysis will be performed on the intention-to-treat (ITT) population, consisting of all patients who were randomized and did not have positive outcomes of the screening tests. We will impute missing data on outcome measures and covariates by multiple imputation, using baseline characteristics and disease activity characteristics of previous study visits known to be a predictor. As sensitivity analysis, a per protocol analysis will be performed, including only patients who will have strictly followed the treatment protocol (including switching treatment). All tests of significance will be performed two-sided with α = 0.05.

#### Secondary endpoints

Secondary endpoints are as follows:

- Repeated continuous outcome measures will be analyzed using a mixed-effects model with adjustment for the same covariates as in the primary analysis, as well as the interaction between time and treatment.
- Binary/categorical data will be compared between the TCZ and prednisone groups and tested for statistical significance using logistic regression analysis, taking the same covariates into account as used in the analysis of the primary outcome.
- Patients who withdraw before 12 months of follow-up because of adverse events or ineffectiveness will be considered as not having reached the effectiveness endpoint in the analysis for binary (response) outcomes.
- Time to event outcomes (drug retention and time to reach remission) will be analyzed using Cox’s proportional hazard regression analysis, with the same covariates being taken into account as used in the analysis of the primary outcome.
- Continuous data will be compared between the two study groups and tested for statistical significance using ANCOVA, with adjustments being made for the same covariates as in the primary analysis. For safety outcomes, we will assess the following endpoints in both groups: the proportion of patients with ≥ 1 SAE, the proportion of patients reaching ≥ 1 of the severest categories of the GTI, and the proportion of patients experiencing ≥ 1 TCZ-associated AE. Differences in statistical significance will be tested using the Cochran-Mantel-Haenszel-test, taking stratification for the center into account.
- Bootstrapping will be combined with single imputation to account for uncertainty in the cost-effectiveness outcomes and missing data in the utility and cost categories [28]. Also cost-effectiveness acceptability planes as well as cost-effectiveness acceptability curves will be presented, thereby graphing the probability that the TCZ strategy is cost-effective compared to using the prednisone strategy as a function of willingness to pay.

### Ethical considerations

We think this study poses a negligible additional risk for patients compared to regular care. The study visits will be performed in combination with the usual visits to the outpatient clinic, and most study assessments would have also been performed in regular care (except for the questionnaires and DEXA-scans). No increased risks are associated with the treatment strategies as they are both used in clinical care for this patient group. Also, the treatment regimens follow the principle of treat-to-target, which implies that the treatment is adjusted when needed (according to predefined disease activity criteria). Moreover, the treating rheumatologist is allowed to deviate from the protocol for medical reasons if necessary in line with usual care. After completion of the study or after discontinuation during the study, patients can continue to use the randomized treatment in regular care. Any unexpected harm caused to patients who are enrolled into the study and that are attributable to the study interventions/assessments will be covered by the insurance of the UMCU. Any harm caused by negligence will be covered by the liability insurance of each participating center. This covers additional health care and compensation.

### Oversight and monitoring

The UMCU is responsible for the design, preparation, and conduct of the entire trial, including dissemination of the results. One rheumatologist at each participating center will be responsible for proper conduct at that site. A data manager at the UMCU is responsible for verification of proper data management during and after the study, and data management will follow a predefined data management plan. An independent monitor will regularly check compliance to the study protocol and current guidelines and regulations. The exact conduct of monitoring is detailed in the monitoring plan (see Additional file 3). In addition, random independent audits on studies in the UMCU are frequently performed. All SAEs that occur during the study will be reported to the medical ethical committee that approved the protocol in line with current legislation. Any future amendment to the protocol will first be reviewed by this committee as well.

## Discussion

The TOPIRA trial will provide valuable insight in the real-world effectiveness, safety, and cost-effectiveness of prednisone versus tocilizumab as addition to csDMARDs in a treat-to-target approach in RA patients with insufficient response to csDMARD therapy alone. The outcomes of this study will reflect the results that can be obtained in regular clinical practice, since the study protocol follows daily care as closely as possible; that is, 1) it leaves space for rheumatologists to change treatment according to the patients’ needs. Should the protocol in certain situations not account for proper clinical care, rheumatologists are at liberty to deviate from the protocol (e.g., not taper medication despite a low CDAI, in case of active disease in the feet, as joint counts used in this study do not assess the feet). 2) The inclusion and exclusion criteria allow for every age group and do not exclude patients with certain comorbidities. 3) Given the pragmatic nature, this is an open-label trial; thus, a placebo/nocebo effect may be present in accordance with clinical practice. However, we also include objective outcome parameters like radiographic progression, which will be scored by an assessor blinded to treatment.

Since follow-up of 1 year does not represent the full extent of toxicity (as well as effectiveness) caused by the long-term use of GCs and TCZ, we are considering a post-trial follow-up period if feasible. The sample size was chosen to be able to show a difference of eight points on the CDAI with a power of 0.80. This is a moderate effect size (Cohen’s *d* = 0.57), and we think TCZ should show at least a moderate additional effect in comparison to prednisone to justify the high drug costs and become the preferred treatment.

In the last 20 years, many new therapeutic agents have been registered for the treatment of RA patients who have insufficient response to csDMARDs alone. Although these agents have all been shown to be efficacious in separate studies, head-to-head trials that directly compare treatment strategies in patients who failed on csDMARDs are sparse [7]. The comparison of bDMARDs with prednisone is of extra interest since they differ significantly in price. This will be the first clinical trial directly comparing the addition of prednisone to the addition of a bDMARD. With the outcomes of this study, we hope to be able to weigh effectiveness, safety, and costs of both treatments in a real-world context.

### Trial status

Protocol version 1.2 from September 20, 2019, has been implemented. This is the first version of the protocol that was approved by the Ethic Committee of the UMC Utrecht. The trial began on January 13, 2020, and is currently recruiting. Recruitment is expected to end on December 31, 2022.

## Supplementary information

**Additional file 1.** CDAI, DAS28 and SDAI formula.

**Additional file 2.** Prednisone taper schedule.

**Additional file 3.** SPIRIT checklist.

**Additional file 4.** EULAR and ACR response criteria.

## Data Availability

The trial protocol is available from the corresponding author on request. An article containing the most important outcomes will be submitted for publication in a peer-reviewed journal within 12 months after availability of the required dataset.

## Abbreviations

ACR: American College of Rheumatology
ALAT: alanine aminotransferase
ASAT: aspartate aminotransferase
CDAI: Clinical Disease Activity Index
CRP: C-reactive protein
DAS28: Disease Activity Score assessing 28 joints
(cs/b/ts)DMARD: (conventional synthetic/ biological/targeted synthetic) disease-modifying antirheumatic drug
ESR: erythrocyte sedimentation rate
EULAR: European League Against Rheumatism
FACIT: Functional Assessment of Chronic Illness Therapy
GC: glucocorticoids
GDA: Global Disease Activity
GTI: Glucocorticoid Toxicity Index
HADS: Hospital Anxiety and Depression Scale
HAQ: Health Assessment Questionnaire
MTX: methotrexate
NSAID: non-steroidal anti-inflammatory drug
PSQI: Pittsburgh Sleep Quality Index
RA: rheumatoid arthritis
SDAI: Simple Disease Activity Index
SJC: Swollen Joint Count
SPIRIT: Standard Protocol Items: Recommendations for Interventional Trials
TCZ: tocilizumab
TJC: Tender Joint Count
TNFi: tumor necrosis factor inhibitor
UMCU: University Medical Center Utrecht
VAS: Visual Analog Scale
