# Supplementary material for: Effectiveness of TOcilizumab in comparison to Prednisone In Rheumatoid Arthritis patients with insufficient response to disease-modifying antirheumatic drugs (TOPIRA): study protocol for a pragmatic trial
Source: Trials. 2020 Apr 5;21:313. doi: 10.1186/s13063-020-04260-y (PMC7133012; doi:10.1186/s13063-020-04260-y)
Supplement: Supplementary file 1 — Additional file 1. CDAI, DAS28 and SDAI formula. [file 13063_2020_4260_MOESM1_ESM.docx]

## Additional file 1: CDAI, DAS28 and SDAI formula

### Clinical Disease Activity Index (CDAI)

Components:

- SJC28: Swollen 28-Joint Count (shoulders, elbows, wrists, metacarpophalangeal joints, proximal interphalangeal joints of hands including thumb interphalangeal joint, knees)
- TJC28: Tender 28-Joint Count (shoulders, elbows, wrists, metacarpophalangeal joints, proximal interphalangeal joints of hands including thumb interphalangeal joint, knees)
- Global Disease Activity on a visual analog scale as assessed by the patient on a scale of 0.0-10.0 (GDA-patient)
- Global Disease Activity on a visual analog scale as assessed by the evaluator on a scale of 0.0-10.0 (GDA-evaluator)

Formula:

$$CDAI=SJC28 + TJC28 +{GDA}_{patient}+ {GDA}_{evaluator}$$

| **CDAI interpretation** | |
| --- | --- |
| Remission | ≤2.8 |
| Low disease activity | 2.9-10.0 |
| Moderate disease activity | 10.1-22.0 |
| High disease activity | 22.1-76.0 |

### Disease Activity Score assessing 28 joints (DAS28)

Components:

- SJC28 and TJC as above
- Erythrocyte sedimentation rate in mm/hour (ESR)
- GDA-patient as above

Formula:

$$DAS28=0.56\sqrt{TJC(28)} + 0.28\sqrt{SJC(28)} +0.70Ln\left( ESR \right) +0.014\times{GDA}_{patient}$$

| **DAS28 interpretation** | |  |
| --- | --- | --- |
| Remission | <2.6 | |
| Low disease activity | ≥2.6 AND ≤3.2 | |
| Moderate disease activity | >3.2 AND ≤5.1 | |
| High disease activity | >5.1 | |

### Simple Disease Activity Index (SDAI)

Components:

- TJC28 and SJC28 as above
- GDA-patient and GDA-evaluator as above
- C-reactive protein (CRP) in mg/dL with the maximum score being 10.0 (i.e. higher concentrations are scored as 10)

Formula:

$$SDAI=TJC\left( 28 \right)+SJC\left( 28 \right)+{GDA}_{patient}+{GDA}_{evaluator}+CRP(units)$$

| **SDAI interpretation** | |
| --- | --- |
| Remission | ≤3.3 |
| Low disease activity | 3.4-11.0 |
| Moderate disease activity | 11.1-26.0 |
| High disease activity | 26.1-86.0 |
