# Supplementary material for: Effectiveness of TOcilizumab in comparison to Prednisone In Rheumatoid Arthritis patients with insufficient response to disease-modifying antirheumatic drugs (TOPIRA): study protocol for a pragmatic trial
Source: Trials. 2020 Apr 5;21:313. doi: 10.1186/s13063-020-04260-y (PMC7133012; doi:10.1186/s13063-020-04260-y)
Supplement: Supplementary file 2 — Additional file 2. Prednisone taper schedule. [file 13063_2020_4260_MOESM2_ESM.docx]

## Additional file 2: Prednisone taper schedule

### Tapering schedule 10mg to 7.5mg

|  | Day 1 | Day 2 | Day 3 | Day 4 | Day 5 | Day 6 | Day 7 |
| --- | --- | --- | --- | --- | --- | --- | --- |
| Week 1-2 | **10 mg** | **10 mg** | **10 mg** | *7.5 mg* | **10 mg** | **10 mg** | **10 mg** |
| Week 3-4 | *7.5 mg* | **10 mg** | **10 mg** | **10 mg** | *7.5 mg* | **10 mg** | **10 mg** |
| Week 5-6 | *7.5 mg* | **10 mg** | **10 mg** | *7.5 mg* | **10 mg** | **10 mg** | *7.5 mg* |
| Week 7-8 | **10 mg** | *7.5 mg* | **10 mg** | *7.5 mg* | **10 mg** | 7.5 mg | **10 mg** |
| Week 9-10 | *7.5 mg* | *7.5 mg* | **10 mg** | *7.5 mg* | *7.5 mg* | **10 mg** | *7.5 mg* |
| Week 11 | *7.5 mg* | *7.5 mg* | **10 mg** | *7.5 mg* | *7.5 mg* | *7.5 mg* | **10 mg** |
| Week 12 | *7.5 mg* | *7.5 mg* | *7.5 mg* | *7.5 mg* | *7.5 mg* | *7.5 mg* | *7.5 mg* |

### Tapering schedule 7.5mg to 5mg

|  | Day 1 | Day 2 | Day 3 | Day 4 | Day 5 | Day 6 | Day 7 |
| --- | --- | --- | --- | --- | --- | --- | --- |
| Week 1-2 | **7.5 mg** | **7.5 mg** | **7.5 mg** | *5 mg* | **7.5 mg** | **7.5 mg** | **7.5 mg** |
| Week 3-4 | *5 mg* | **7.5 mg** | **7.5 mg** | **7.5 mg** | *5 mg* | **7.5 mg** | **7.5 mg** |
| Week 5-6 | *5 mg* | **7.5 mg** | **7.5 mg** | *5 mg* | **7.5 mg** | **7.5 mg** | *5 mg* |
| Week 7-8 | **7.5 mg** | *5 mg* | **7.5 mg** | *5 mg* | **7.5 mg** | 5 mg | **7.5 mg** |
| Week 9-10 | *5 mg* | *5 mg* | **7.5 mg** | *5 mg* | *5 mg* | **7.5 mg** | *5 mg* |
| Week 11 | *5 mg* | *5 mg* | **7.5 mg** | *5 mg* | *5 mg* | *5 mg* | **7.5 mg** |
| Week 12 | *5 mg* | *5 mg* | *5 mg* | *5 mg* | *5 mg* | *5 mg* | 1. *mg* |
