# Supplementary material for: Effectiveness of TOcilizumab in comparison to Prednisone In Rheumatoid Arthritis patients with insufficient response to disease-modifying antirheumatic drugs (TOPIRA): study protocol for a pragmatic trial
Source: Trials. 2020 Apr 5;21:313. doi: 10.1186/s13063-020-04260-y (PMC7133012; doi:10.1186/s13063-020-04260-y)
Supplement: Supplementary file 3 — Additional file 3. SPIRIT checklist. [file 13063_2020_4260_MOESM3_ESM.pdf]

SPIRIT 2013 Checklist: Recommended items to address in a clinical trial protocol and related documents*

Section/item	ItemNo	Description
**Administrative information**		
Title	1	*Effectiveness of TOcilizumab in comparison to Prednisone In Rheumatoid Arthritis patients with insufficient response to disease modifying anti-rheumatic drugs (TOPIRA): study protocol for a pragmatic trial.*
Trial registration	2a	*Page 3: trial registration*
	2b	*Page 3: trial registration*

Data category	Information
Primary registry and trial identifying number	Netherlands Trial Register: NL8070
Date of registration in primary registry	October 7, 2019
Secondary identifying numbers	ABR: NL68492.041.19<br>EUDRA-CT: 2017-003037-28<br>METC: 19/419
Source(s) of monetary or material support	University Medical Center Utrecht and Hoffmann-La Roche
Primary sponsor	University Medical Center Utrecht (UMCU)
Secondary sponsor(s)	Not applicable, the UMCU is the only sponsor of this trial.
Contact for public queries	*M.S. van der Leeuw, MD*. University Medical Center Utrecht.<br>m.s.vanderleeuw-17@umcutrecht.nl
Contact for scientific queries	*M.S. van der Leeuw, MD*. University Medical Center Utrecht.
Public title	Efficacy of TOcilizumab in comparison to Prednisone In Rheumatoid Arthritis patients with insufficient response to disease modifying anti-rheumatic drugs (TOPIRA)
Scientific title	Efficacy of TOcilizumab in comparison to Prednisone In Rheumatoid Arthritis patients with insufficient response to disease modifying anti-rheumatic drugs (TOPIRA)
Countries of recruitment	The Netherlands
Health condition(s) or problem(s) studied	Rheumatoid Arthritis

Intervention(s)		Intervention 1: Tocilizumab subcutaneously 162mg/week<br>Intervention 2: Prednisone orally 10mg/day in combination with vitamin D 800IU/day, calcium 1000mg/day and alendronic acid 70 mg/week
Key inclusion and exclusion criteria		This is addressed on pages 8-9 of the manuscript.
Study type		Interventional<br>Allocation: randomized<br>Intervention model: parallel assignment<br>Open-label<br>Primary purpose: treatment<br>Phase IV
Date of first enrolment		February 2020
Target sample size		120
Recruitment status		Recruiting
Primary outcome(s)		Difference in Clinical Disease Activity Score (CDAI) from baseline to 12 months.
Key secondary outcomes		Radiographic progression, drug retention and compliance rates, DAS28, SDAI, remission rates, functional ability, fatigue, sleep quality, anxiety and depression, cost-effectiveness, pain, GC-associated AEs, TCZ-associated AEs, SAEs.
Protocol version	3	*This is addressed on page 21 of the manuscript under the heading 'Trial Status'*
Funding	4	*This is addressed on page 23 of the manuscript under the heading 'Funding'*
Roles and responsibilities	5a	*This is addressed on page 24 of the manuscript under the heading 'Authors' contributions'*
	5b	*This is addressed on page 4 of the manuscript under the heading 'Administrative information'*
	5c	*This is addressed on page 23 of the manuscript under the heading 'Funding'*
	5d	*This is addressed on pages 20 of the manuscript under the subheading 'Oversight and monitoring'*
**Introduction**		
Background and rationale	6a	*This is addressed on pages 5-7 of the manuscript under the heading Background*
	6b	*This is addressed on pages 5-7 of the manuscript under the heading Background*
Objectives	7	*This is addressed on page 7 of the manuscript under the subheading Objectives*
Trial design	8	*This is addressed on page 10 of the manuscript under the subheading Study Design*
Methods: Participants, interventions, and outcomes		

Study setting	9	*This is addressed on page 8 of the manuscript under the headings 'Methods'*
Eligibility criteria	10	*This is addressed on pages 8-9 of the manuscript under the subheadings Inclusion criteria and Exclusion criteria.*
Interventions	11a	*This is addressed on pages 8-13 of the manuscript under the headings 'Methods'*
	11b	*This is addressed on pages 8-13 of the manuscript under the heading 'Methods'*
	11c	*This is addressed on page 15 of the manuscript under the subheading 'Secondary endpoints'*
	11d	*This is addressed on page 11-12 of the manuscript under the subheading 'Treatment strategies'*
Outcomes	12	*This is addressed on page 14-17 of the manuscript under the subheading 'Endpoints'*
Participant timeline	13	*The timeline is described in the SPIRIT figure. This Figure will be included on page 17 of the manuscript. It is uploaded as a separate file.*
Sample size	14	*This is addressed on page 9-10 of the manuscript under the subheading 'Sample Size'*
Recruitment	15	*This is addressed on page 8 of the manuscript under the heading 'Methods'*<br>The enrollment period is anticipated to be 2 years *(this is addressed on page 21 under the heading trial status).*
Methods: Assignment of interventions (for controlled trials)		
Allocation:		
Sequence generation	16a	*This is addressed on pages 10-11 of the manuscript under the subheading 'Study design'*
Allocation concealment mechanism	16b	*This is addressed on pages 10-11 of the manuscript under the subheading 'Study design'*
Implementation	16c	*This is addressed on page 8 of the manuscript under the heading 'Methods' and on page 10 under the subheading 'Study design'*
Blinding (masking)	17a	Treatment will not be blinded. *This is addressed on page 8 of the manuscript under the heading 'Methods'*
	17b	Not applicable, the study is not blinded.
Methods: Data collection, management, and analysis		
Data collection methods	18a	*This is addressed on page 8 of the manuscript under the heading 'Methods'*
	18b	*This is addressed on pages 12-13 of the manuscript under the subheading 'Withdrawal'*
Data management	19	*This is addressed on page 8 of the manuscript under the heading 'Methods'*

Statistical methods	20a	*This is addressed on pages 18-19 of the manuscript under the subheading 'Statistical Analyses'*
	20b	Not applicable, we do not plan on performing additional analyses.
	20c	*This is addressed on pages 18-19 of the manuscript under the subheading 'Statistical Analyses'*
Methods: Monitoring		
Data monitoring	21a	*This is addressed on pages 20 of the manuscript under the subheading 'Oversight and monitoring'*
	21b	Not applicable, no interim analyses will be performed.
Harms	22	*This is addressed on page 15-16 of the manuscript under the subheading 'Safety Outcomes' and pages 19-20 under the subheading 'Ethical considerations' and page 20 under the heading 'Oversight and monitoring'*
Auditing	23	*This is addressed on pages 20 of the manuscript under the subheading 'Oversight and monitoring'*
Ethics and dissemination		
Research ethics approval	24	*This is addressed on page 23 of the manuscript under the subheading 'Approvals'*
Protocol amendments	25	*This is addressed on page 20 of the manuscript under the subheading 'Oversight and monitoring'*
Consent or assent	26a	*This is addressed on page 8 of the manuscript under the heading 'Methods'*
	26b	*This is addressed on page 8 of the manuscript under the heading 'Methods'*
Confidentiality	27	*This is addressed on page 8 of the manuscript under the heading 'Methods'*
Declaration of interests	28	*This is addressed on page 23 of the manuscript under the heading 'Competing interests'*
Access to data	29	*This is addressed on page 23 of the manuscript under the heading 'Availability of data and materials'*
Ancillary and post-trial care	30	*This is addressed on pages 19-20 of the manuscript under the subheading 'Ethical considerations'*
Dissemination policy	31a	*This is addressed on page 23 of the manuscript under the heading 'Availability of data and materials'*
	31b	*This is addressed on page 24 of the manuscript under the heading 'Authors' contributions'*
	31c	*This is addressed on page 23 of the manuscript under the heading 'Availability of data and materials'*
**Appendices**		
Informed consent materials	32	See appendix for consent form and information folder (in Dutch)

Biological specimens	33	Not applicable. No specimens will be stored for this study. The laboratory assessments are performed in regular care, and the specimens used for these assessments will not be stored for later analysis.
Monitoring plan		See appendix for the monitoring plan (in Dutch).

*It is strongly recommended that this checklist be read in conjunction with the SPIRIT 2013 Explanation & Elaboration for important clarification on the items. Amendments to the protocol should be tracked and dated. The SPIRIT checklist is copyrighted by the SPIRIT Group under the Creative Commons "Attribution-NonCommercial-NoDerivs 3.0 Unported" license.

APPENDIX: patient information folder and informed consent form (Dutch)

De effectiviteit van TOcilizumab in vergelijking met PrednIson bij patiënten met Reumatoïde Artritis (TOPIRA)

Geachte heer/mevrouw,

Wij vragen u om mee te doen aan een medisch-wetenschappelijk onderzoek. Meedoen is vrijwillig. Om mee te doen is wel uw schriftelijke toestemming nodig. Voordat u beslist of u wilt meedoen aan dit onderzoek, krijgt u uitleg over wat het onderzoek inhoudt. Lees deze informatie rustig door en vraag de onderzoeker uitleg als u vragen heeft. U kunt ook de onafhankelijk deskundige, die aan het eind van deze brief genoemd wordt, om aanvullende informatie vragen. U kunt er ook over praten met uw partner, vrienden of familie. Verdere informatie over meedoen aan zo'n onderzoek staat in de bijgevoegde brochure 'Medisch-wetenschappelijk onderzoek'.

1. Algemene informatie
Dit onderzoek is opgezet door de afdeling Reumatologie & Klinische Immunologie van het UMC Utrecht en wordt uitgevoerd door artsen en onderzoekers in verschillende ziekenhuizen. Aan dit onderzoek zullen 120 patiënten meedoen.
De medisch-ethische toetsingscommissie van het UMC Utrecht heeft dit onderzoek goedgekeurd. Algemene informatie over de toetsing van onderzoek vindt u in bijgevoegde brochure 'Medisch-wetenschappelijk onderzoek'.

2. Doel van het onderzoek
Het doel van het onderzoek is uitzoeken of behandeling met tocilizumab effectiever is dan een behandeling met prednison, en of dit tot minder bijwerkingen leidt.

3. Achtergrond van het onderzoek
Als u bent gevraagd om deel te nemen aan dit onderzoek, bent u in het verleden al met meerdere reumaremmende medicijnen behandeld. Toch blijft u op dit moment last houden van uw gewrichten. Er zijn in dit geval verschillende medicijnen beschikbaar om toe te voegen aan uw huidige medicatie, zodat de reuma beter behandeld kan worden. Op dit moment is het niet duidelijk welk van deze medicijnen effectiever en veiliger is. Er bestaat daarom geen standaardbehandeling voor patiënten zoals u. In deze studie willen we twee van de beschikbare middelen met elkaar vergelijken: prednison en tocilizumab. Beide middelen worden in de praktijk ook al gebruikt om patiënten te behandelen die onvoldoende reageren op eerdere medicijnen.
Tocilizumab is een biological, gericht tegen het ontstekingseiwit IL-6. IL-6 wordt aangemaakt bij ontstekingsprocessen en houdt ontsteking in stand. Tocilizumab remt de werking van IL-6. Het wordt wekelijks met onderhuidse injecties gegeven.
Prednison is een medicijn dat ontstekingsverschijnselen van allerlei ziekten, ook RA, kan remmen. Prednison wordt dagelijks gegeven in de vorm van een tablet. Tijdens dit onderzoek wordt prednison voorgeschreven in de veel gebruikte (lage) dosering van 10mg/dag.
Beide middelen kunnen gewrichtsontstekingen verminderen en schade aan gewrichten beperken, maar beide kunnen ook bijwerkingen veroorzaken. Het is onbekend of tocilizumab effectiever is dan prednison om de ziekte te onderdrukken. Ook weten we niet of er minder bijwerkingen optreden dan bij behandeling met prednison.

4. Wat meedoen inhoudt
Als u meedoet, duurt dat in totaal ongeveer 12 maanden voor u.

A. Geschiktheidsbezoek

Eerst bepalen we of u kunt meedoen. De onderzoeker doet lichamelijk onderzoek, noteert geslacht en leeftijd en vraagt naar uw medische geschiedenis. Ook bepalen we de ziekteactiviteit aan de hand van gewrichtsonderzoek. Dit is niet anders dan in de gebruikelijke zorg. Daarnaast nemen we bloed af om te onderzoeken of er sprake is van hepatitis B en C en maken we een röntgenfoto van de longen om te onderzoeken of er sprake is van tuberculose. Dit zijn allemaal infectieziekten. Deze onderzoeken doen we altijd bij patiënten die starten met tocilizumab.

B. Behandeling

Als u na het geschiktheidsbezoek in aanmerking komt voor deelname, blijft u de klassieke reumaremmende medicijnen doorgebruiken.

<u>Loting bepaalt welke aanvullende behandeling u krijgt</u>: de helft van de deelnemers krijgt tocilizumab (162mg per week) erbij, de andere helft krijgt prednison (10mg per dag) erbij. Dit zijn de doseringen die ook in de reguliere praktijk gebruikt worden.

Patiënten die prednison krijgen, zullen daarnaast alendroninezuur (70mg/week) en calcium (1000mg) met vitamine D (800IE/dag) krijgen ter bescherming van hun botten. Dit is standaard bij behandeling met prednison.

Als tijdens de behandeling de ziekteactiviteit gedurende minstens 3 maanden rustig is, zal de medicatie worden afgebouwd:

- Voor patiënten die tocilizumab krijgen, wordt de tocilizumab dan om de week gebruikt in plaats van wekelijks. Mocht de ziekte dan weer opvlammen, gaat de dosering terug naar één keer per week. Als daarna de ziekte nog steeds actief is, stoppen we de tocilizumab en zult u behandeld worden met prednison.
- Voor patiënten die prednison krijgen, wordt de dosis van 10mg verlaagd naar 7.5mg en vervolgens naar 5mg. Mocht de ziekte weer opvlammen tijdens het afbouwen, dan wordt de dosering 7.5mg of 10mg, afhankelijk van de dosering op het moment van opvlammen van de ziekte. De dosering wordt maximaal weer opgebouwd naar 10mg/dag. Als daarna de ziekte nog steeds actief is, stoppen we de prednison en zult u behandeld worden met tocilizumab.

<u>Als vanaf 2 maanden na starten met de behandeling de ziekteactiviteit te hoog blijft</u>, zullen patiënten de behandeling van de andere groep ontvangen. Diegenen die in eerst behandeld werden met tocilizumab krijgen dan prednison, en andersom. Het kan ook zo zijn dat uw reumatoloog vindt dat u de medicatie moet stoppen wegens bijwerkingen. In dat geval krijgt u ook de behandeling van de andere groep. Mocht de ziekteactiviteit dan alsnog te hoog blijven of moet u de behandeling stoppen wegens andere medische redenen, dan is de keuze voor een behandeling vrij en overlegt u samen met uw reumatoloog over de vervolgbehandeling. U brengt dan nog wel iedere 3 maanden een bezoek aan het ziekenhuis tot en met 12 maanden na start van deelname aan de studie.

C. Bezoeken en metingen

Voor het onderzoek is het nodig dat u de eerste 3 maanden elke maand naar het ziekenhuis komt. Hierna komt u om de 3 maanden naar het ziekenhuis. Dat is in totaal 8 keer in 12 maanden (inclusief het geschiktheidsbezoek en het eerste bezoek). Deze bezoeken zijn dezelfde als uw normale controlebezoeken aan de reumatoloog, alleen doen we een aantal extra onderzoekshandelingen.

De volgende handelingen gebeuren standaard bij een bezoek aan de reumatoloog, ook als u niet aan dit onderzoek mee doet:

- Onderzoek van de gewrichten
- Bloedonderzoek. De bloedbezinking en het CRP (beiden maten van ontsteking), evenals het aantal witte bloedcellen, nier- en leverwaarden, bloedglucose en LDL-cholesterol worden bepaald. Wel wordt er bij dit onderzoek de eerste keer iets meer bloed afgenomen dan normaal om reumafactoren en anti-CCP te bepalen.
- Meten van gewicht, bloeddruk, hartslag
- Voor patiënten die behandeld gaan worden met tocilizumab: eenmalig screening op infectieziekten met een bloedtest voor Hepatitis B, Hepatitis C en tuberculose, inclusief een röntgenfoto van de longen.

Tijdens die bezoeken doen we de volgende extra onderzoekshandelingen:

- Meten van lengte
- Voor patiënten die behandeld gaan worden met prednison: eenmalig screening op infectieziekten met een bloedtest voor Hepatitis B, Hepatitis C en tuberculose, inclusief een röntgenfoto van de longen. Dit is extra voor dit onderzoek, omdat normaal gesproken niet iedereen die prednison gebruikt op deze ziektes gescreend wordt.Zwangerschapstest voor vrouwen in de vruchtbare levensfase met bloed- of urineonderzoek (dit wordt gelijktijdig met de screening op infectieziekten afgenomen).
- Röntgenfoto's van beide handen en voeten (alleen bij het eerste en laatste bezoek).
- Botdichtheidsmeting (DEXA-onderzoek), om de invloed van de behandelingen op de botdichtheid te vergelijken (alleen bij het eerste en laatste bezoek).

We vragen naar:

- Eventuele bijwerkingen (specifiek voor tocilizumab en prednison).
- Uw rookgedrag en alcoholgebruik.
- Mocht u tussendoor last van bijwerkingen krijgen of veel last van uw gewrichten, dan plannen we uiteraard een extra bezoek in.

Ook vragen we u vragenlijsten in te vullen. Die gaan over:

- Dagelijks functioneren
- Kwaliteit van leven
- Angst en depressie, vermoeidheid en verminderde kwaliteit van slapen. Dit zijn factoren die bij veel RA patiënten een rol spelen.
- Kosten die u gemaakt hebt in relatie tot uw gezondheid.

De vragenlijsten kunt u thuis via internet invullen. U ontvangt hiervoor bij ieder bezoek een e-mail met een link naar de vragenlijsten. Uw emailadres zal niet met anderen dan uw behandelend arts en/of verpleegkundige worden gedeeld. Als u niet in de gelegenheid bent om deze vragenlijsten online in te vullen, kunt u de

vragenlijsten op de polikliniek op papier invullen. Het invullen van deze vragenlijsten zal ongeveer 20 tot 30 minuten per keer in beslag nemen.

In bijlage C ‘Schema onderzoekshandelingen’ vindt u een schematisch overzicht waarin staat wanneer welke onderzoekshandelingen plaatsvinden.

D. HandScan onderzoek

Sommige ziekenhuizen waar het TOPIRA onderzoek wordt uitgevoerd, hebben beschikking over een HandScan. In deze ziekenhuizen wordt aanvullend het HandScan onderzoek uitgevoerd. Indien dit in uw behandelcentrum plaatsvindt, ontvangt u hiervoor een aparte informatiebrief en een apart toestemmingsformulier. U kunt vrijwillig aan dit aanvullende onderzoek deelnemen. Als u niet deelneemt aan dit HandScan onderzoek, kunt u toch deelnemen aan het hoofdonderzoek.

E. Anders dan bij gebruikelijke zorg

Het geschiktheidsbezoek gebeurt extra, dus *naast* uw bezoeken aan het ziekenhuis voor uw gebruikelijke zorg. Alle andere bezoeken gebeuren *in* de gebruikelijke zorg. Dit betekent dat de onderzoekshandelingen plaatsvinden tijdens uw controlebezoeken in het ziekenhuis. Wel duren deze bezoeken maximaal 15 minuten langer.
Voor het onderzoek wordt niet vaker bloed afgenomen dan in de gebruikelijke zorg. Wel wordt er bij het eerste bezoek een iets grotere hoeveelheid bloed per keer afgenomen (maximaal 15 ml extra). Het invullen van vragenlijsten en het maken van röntgenfoto’s gebeurt speciaal voor het onderzoek.

5. Wat wordt er van u verwacht

Om het onderzoek goed te laten verlopen, verwachten wij dat u zich aan de volgende afspraken houdt.

De afspraken zijn dat u:

- De medicijnen gebruikt zoals met u besproken;
- De klassieke reumaremmende medicijnen, die u bij aanvang van het onderzoek al gebruikt, op dezelfde manier door blijft gebruiken. Zo worden de resultaten niet beïnvloed door veranderingen in het gebruik van deze klassieke reumaremmende medicijnen;
- Niet ook nog aan een ander medisch-wetenschappelijk onderzoek meedoet;
- Afspraken voor bezoeken nakomt;
- De deelnemerskaart van het onderzoek bij u draagt. Hierop staat dat u meedoet aan dit onderzoek. Er staat ook op wie in geval van nood moet worden gewaarschuwd. Laat deze kaart zien als u bij een andere arts komt.

Het is belangrijk dat u contact opneemt met de onderzoeker:

- Voordat u andere geneesmiddelen voor uw reuma gaat gebruiken;
- Als u in een ziekenhuis wordt opgenomen of behandeld;
- Als u plotseling gezondheidsklachten krijgt;
- Als u niet meer wilt meedoen aan het onderzoek;
- Als uw contactgegevens wijzigen.

F. Zwangerschap

Vrouwen die tijdens de studieperiode zwanger willen worden, zwanger zijn of borstvoeding geven, kunnen niet meedoen aan dit onderzoek. Zwangere vrouwen mogen namelijk geen botdichtheidsscan ondergaan. Ook is het niet duidelijk of tocilizumab veilig gebruikt kan worden tijdens de zwangerschap. Vrouwen in de vruchtbare leeftijd moeten daarom geschikte voorbehoedsmiddelen gebruiken gedurende het hele onderzoek, en tot minstens 3 maanden na staken van behandeling met tocilizumab. De onderzoeker praat met u over geschikte voorbehoedsmiddelen. Vrouwen in de vruchtbare levensfase die meedoen aan dit onderzoek krijgen vooraf een zwangerschapstest.
Wordt u tijdens het onderzoek toch zwanger? Laat dit dan direct weten aan de onderzoeker en uw reumatoloog. De zwangerschap kan dan extra gecontroleerd worden. Dit is niet anders dan in de gebruikelijke zorg.

6. Mogelijke bijwerkingen en andere ongemakken

De medicijnen waarmee u in het onderzoek behandeld wordt, kunnen bijwerkingen hebben. Deze staan beschreven in bijgevoegde informatiefolders van de betreffende medicijnen. Dit is niet anders dan in de gebruikelijke zorg. Als u een mogelijke bijwerking hebtt zal uw reumatoloog beoordelen of het medisch verantwoord is om door te gaan met de medicijnen.

G. Metingen

Voor het onderzoek wordt niet vaker bloed afgenomen dan in de gebruikelijke zorg. Wel wordt er bij het eerste bezoek een iets grotere hoeveelheid bloed per keer afgenomen (maximaal 15ml extra). Bloedafnames kunnen pijn doen of een bloeduitstorting geven. Dit is niet anders dan in de gebruikelijke zorg.

H. Stralingsbelasting

Bij röntgenfoto's en de botdichtheidsmeting maken we gebruik van röntgenstraling. De totale stralingsbelasting in deze onderzoeken is 0,08 mSv. Ter vergelijking: de achtergrondstraling in Nederland is ~2,5 mSv per jaar. De kans is klein dat de straling die tijdens het onderzoek wordt gebruikt, kan leiden tot schade aan uw gezondheid.

7. Mogelijke voor- en nadelen

Het is belangrijk dat u de mogelijke voor- en nadelen goed afweegt voordat u besluit mee te doen.
U hebt zelf geen voordeel van meedoen aan dit onderzoek. Uw deelname kan wel bijdragen aan toekomstige behandeling van patiënten met RA en verbetering van de gezondheidszorg.

Deelname aan het onderzoek betekent:

- Iets langere bezoeken aan het ziekenhuis;
- (Extra) metingen met mogelijke ongemakken;
- Dat we afspraken maken, waaraan u zich moet houden.

Al deze zaken zijn in hoofdstukken 4, 5 en 6 beschreven.

8. Als u niet wilt meedoen of wilt stoppen met het onderzoek

U beslist zelf of u meedoet aan het onderzoek. Deelname is vrijwillig. Als u niet wilt meedoen, wordt u op de gebruikelijke manier behandeld voor uw ziekte. Uw reumatoloog zal de mogelijke behandelingen met u bespreken.

Als u wel meedoet, kunt u zich altijd bedenken en toch stoppen, ook tijdens het onderzoek. U wordt dan weer op de gebruikelijke manier behandeld voor uw ziekte. U hoeft niet te zeggen waarom u stopt. Wel moet u dit direct melden aan de onderzoeker. De gegevens die tot dat moment zijn verzameld, worden gebruikt voor het onderzoek.

Het is ook mogelijk dat u wilt stoppen met de medicatie van het onderzoek, maar nog wel bereid bent de bezoeken en bijbehorende onderzoeken te ondergaan. Als u hiermee instemt dan blijft u deelnemer van het onderzoek en zullen we uw gegevens blijven verzamelen. De behandeling kunt u dan verder bespreken met uw reumatoloog.

Als er nieuwe informatie over het onderzoek bekend wordt die belangrijk voor u is, laat de onderzoeker dit aan u weten.

9. Einde van het onderzoek

Uw deelname aan het onderzoek stopt als:

- Alle bezoeken voorbij zijn;
- U zelf kiest om te stoppen;
- U zwanger wordt;
- De onderzoeker of uw reumatoloog het beter voor u vindt om te stoppen;
- het UMC Utrecht, de overheid of de beoordelende medisch-ethische toetsingscommissie, besluit om het onderzoek te stoppen.

Het hele onderzoek is afgelopen als alle deelnemers klaar zijn. De medicatie die u gebruikt hebt bij het onderzoek, kunt u na afloop van het onderzoek blijven gebruiken, indien gewenst. Uw reumatoloog zal met u de verdere medische zorg bespreken.

10. Gebruik en bewaren van uw gegevens

Voor dit onderzoek worden uw persoonsgegevens verzameld, gebruikt en bewaard. Het gaat om gegevens zoals uw naam en om gegevens over uw gezondheid. Het verzamelen, gebruiken en bewaren van uw gegevens is nodig om onze onderzoeksvragen te kunnen beantwoorden en de resultaten te kunnen publiceren. Wij vragen voor het gebruik van uw gegevens uw toestemming.

I. Vertrouwelijkheid van uw gegevens

Om uw privacy te beschermen krijgen uw gegevens een code. Uw naam en andere gegevens die u direct kunnen identificeren, laten we hierbij weg. Alleen met de sleutel van de code zijn gegevens tot u te herleiden. De sleutel van de code blijft veilig opgeborgen in de onderzoekslocatie (uw eigen behandelcentrum). De gegevens die naar eventuele andere betrokken partijen worden gestuurd bevatten alleen de code, maar niet uw naam of andere gegevens waarmee u kunt worden geïdentificeerd. Ook in rapporten en publicaties over het onderzoek zijn de gegevens niet tot u te herleiden.

J. Toegang tot uw gegevens voor controle

Sommige personen kunnen op de onderzoekslocatie toegang krijgen tot al uw gegevens. Ook tot de gegevens zonder code. Dit is nodig om te kunnen controleren of het onderzoek goed en betrouwbaar is uitgevoerd. Personen die ter controle inzage krijgen in uw gegevens zijn: de commissie die de veiligheid van het onderzoek in de gaten houdt, een monitor die voor de opdrachtgever van het onderzoek werkt, nationale en internationale toezichthoudende autoriteiten, bijvoorbeeld, de Inspectie Gezondheidszorg en Jeugd. Zij houden uw gegevens geheim. Wij vragen u voor deze inzage toestemming te geven.

K. Bewaartermijn gegevens

Uw gegevens moeten 15 jaar worden bewaard op de onderzoekslocatie (uw eigen ziekenhuis of behandelcentrum) en het UMC Utrecht.

L. Bewaren en gebruik van gegevens voor ander onderzoek

Uw gecodeerde gegevens kunnen na afloop van dit onderzoek ook nog van belang zijn voor ander wetenschappelijk onderzoek op het gebied van uw aandoening en/of van de verdere ontwikkeling van de behandelmethode. U kunt op het toestemmingsformulier aangeven of u hier wel of niet mee instemt. Indien u hier niet mee instemt, kunt u gewoon deelnemen aan het huidige onderzoek.

M. Informatie over onverwachte bevindingen

Tijdens dit onderzoek kan er bij toeval iets gevonden worden dat niet van belang is voor het onderzoek maar wel voor U. Als dit belangrijk is voor uw gezondheid, dan brengen wij u, uw huisarts en/of behandelend reumatoloog op de hoogte. U kunt dan met uw huisarts of reumatoloog bespreken wat er moet gebeuren. Ook hiervoor geeft u toestemming.

N. Intrekken toestemming

U kunt uw toestemming voor gebruik van uw persoonsgegevens altijd weer intrekken. Dit geldt voor dit onderzoek en ook voor het bewaren en het gebruik voor het toekomstige onderzoek. De onderzoeksgegevens die zijn verzameld tot het moment dat u uw toestemming intrekt worden nog wel gebruikt in het onderzoek.

O. Doorgifte naar landen buiten de Europese Unie (EU)

Indien u toestemming geeft dat uw gegevens na afloop van dit onderzoek gebruikt kunnen worden voor ander wetenschappelijk onderzoek op het gebied van uw aandoening en/of van de verdere ontwikkeling van de behandelmethode, kan het zijn dat uw gecodeerde gegevens ook worden doorgestuurd naar het bedrijf Roche in Zwitserland, gelegen buiten de EU. Dit bedrijf financiert voor een groot deel dit TOPIRA onderzoek. De EU heeft vastgesteld dat in Zwitserland een gelijkwaardig niveau van gegevensbescherming geldt. Uw privacy zal dan op een gelijkwaardig niveau worden beschermd.

P. Meer informatie over uw rechten bij verwerking van gegevens

Voor algemene informatie over uw rechten bij verwerking van uw persoonsgegevens kunt u de website van de Autoriteit Persoonsgegevens raadplegen. Bij vragen over uw rechten kunt u contact opnemen met de verantwoordelijke voor de verwerking van uw persoonsgegevens. Voor dit onderzoek is dat het UMC Utrecht. Zie bijlage A voor contactgegevens en website.

Bij vragen of klachten over de verwerking van uw persoonsgegevens raden we u aan eerst contact op te nemen met uw eigen behandelcentrum. U kunt ook contact opnemen met de Functionaris voor de Gegevensbescherming van het UMC Utrecht (zie contactgegevens in bijlage A) of de Autoriteit Persoonsgegevens.

Q. Registratie van het onderzoek

Informatie over dit onderzoek is ook opgenomen in een overzicht van medisch-wetenschappelijke onderzoeken namelijk www.clinicaltrials.gov. Daarin zijn geen gegevens opgenomen die naar u herleidbaar zijn. Na het onderzoek kan de website een samenvatting van de resultaten van dit onderzoek tonen. U vindt dit onderzoek onder de naam ‘TOPIRA’.

11. Verzekering voor deelnemers
Voor iedereen die meedoet aan dit onderzoek is een verzekering afgesloten. De verzekering dekt schade door het onderzoek. Niet alle schade is gedekt. In bijlage B 'Informatie over de verzekering' vindt u meer informatie over de verzekering. Daar staat ook beschreven aan wie u schade kunt melden.

12. Informeren huisarts en apotheker
Wij sturen uw huisarts altijd een brief om te laten weten dat u meedoet aan het onderzoek. Dit is voor uw eigen veiligheid. De apotheek waar u de medicatie voor dit onderzoek ophaalt, zal geïnformeerd worden over de medicatie die u gebruikt. In sommige gevallen kan dit een andere apotheek zijn dan uw eigen apotheek. Als u dit niet goed vindt, kunt u niet meedoen aan dit onderzoek.

13. Vergoeding onkosten
De behandelingen gebeuren via de gebruikelijke zorg. Aan alle handelingen die in de gebruikelijke zorg niet zouden zijn gedaan (dus specifiek voor het onderzoek gebeuren), zijn voor u geen kosten verbonden. U wordt niet betaald voor het meedoen aan dit onderzoek. Wel krijgt u een vergoeding voor extra reiskosten voor bezoeken aan het ziekenhuis die u niet gemaakt zou hebben in de gebruikelijke zorg. In dit onderzoek geldt dat alleen voor het eerste geschiktheidsbezoek en eventuele onverwachte extra bezoeken vanwege het onderzoek.

14. Hebt u vragen?
Bij vragen kunt u contact opnemen met het onderzoeksteam. Voor onafhankelijk advies over meedoen aan dit onderzoek kunt u terecht bij de onafhankelijke arts. Zij weet veel over het onderzoek, maar heeft niets te maken met dit onderzoek.
Bij klachten kunt u het beste terecht bij de klachtencommissie van het ziekenhuis waar u deelneemt. Alle gegevens vindt u in bijlage A 'Contactgegevens'.

15. Ondertekening toestemmingsformulier
Wanneer u voldoende bedenktijd heeft gehad, wordt u gevraagd te beslissen over deelname aan dit onderzoek. Indien u toestemming geeft, zullen wij u vragen deze op de bijbehorende toestemmingsverklaring schriftelijk te bevestigen. Door uw schriftelijke toestemming geeft u aan dat u de informatie heeft begrepen en instemt met deelname aan het onderzoek. Zowel uzelf als de onderzoeker ontvangen een getekende versie van deze toestemmingsverklaring.

Dank voor uw aandacht.

16. Bijlagen bij deze informatiebrief
A. Contactgegevens
B. Informatie over de verzekering
C. Schema onderzoekshandelingen
D. Toestemmingsformulier deelnemer
E. Brochure 'Medisch-wetenschappelijk onderzoek. Algemene informatie voor de proefpersoon' (versie februari 2019)
F. Informatiefolder Prednison
G. Informatiefolder Tocilizumab

17. Belangenverstrengeling en financiering van het onderzoek
Dit onderzoek is een initiatief vanuit reumatologen in het UMC Utrecht. Het onderzoek is opgezet in een samenwerkingsverband tussen reumatologieafdelingen in Nederland en het bedrijf Roche. Het bedrijf Roche is de financier van dit onderzoek. De onderzoekers verklaren dat zij geen functies bekleden die voor dit onderzoek kunnen leiden tot tegenstrijdige belangen.

Apart als PDF

Bijlage A: Contactgegevens voor UMC Utrecht

Coördinerend Arts-Onderzoeker:
Dhr. Matthijs van der Leeuw
E-mailadres: topira@umcutrecht.nl
Telefoonnummer: 088 75 517 70

Hoofd-Onderzoeker:
Prof. Dr. J.M. van Laar
Telefoonnummer: 088 75 573 57

Onderzoeksverpleegkundigen:
Mw. A. Cirkel, Mw. J. Nijdeken, Mw. K. Schrijvers
E-mailadres: reumatologie-research@umcutrecht.nl
Telefoonnummer: 088 75 504 35

Onafhankelijk arts:
Dr. H. Leavis, Internist-Immunoloog, UMC Utrecht
Telefoonnummer: 088 75 573 57

Klachten over het onderzoek:
Als u klachten hebt, kunt u dit melden aan de onderzoeker of aan uw behandelend arts. Mocht u ontevreden zijn over de gang van zaken bij het onderzoek en een klacht willen indienen dan kunt u contact opnemen met de klachtenbemiddelaars. Deze zijn bereikbaar via tel. 088-755 62 08.
Of digitaal via: http://www.umcutrecht.nl/nl/Ziekenhuis/Ervaringen-van-patienten/Een-klacht-indienen.

Klachten over verwerking persoonsgegevens:
Indien u vragen of klachten heeft over de verwerking van uw persoonsgegevens raden we u aan eerst contact op te nemen met de onderzoeker of uw behandelend arts. U kunt ook contact opnemen met de Functionaris voor de Gegevensbescherming. Deze is bereikbaar via e-mailadres privacy@umcutrecht.nl.Op de corporate website https://www.umcutrecht.nl/nl/Ziekenhuis/In-het-ziekenhuis/Regels-en-rechten/Rechten staat informatie over uw rechten.

Bijlage B: Informatie over de verzekering

Voor iedereen die meedoet aan dit onderzoek is een verzekering afgesloten. De verzekering dekt schade door deelname aan het onderzoek. Dit geldt voor schade tijdens het onderzoek of binnen vier jaar na het einde van uw deelname aan het onderzoek. Schade moet u binnen die vier jaar aan de verzekeraar hebben gemeld.

De verzekering dekt niet alle schade. Onderaan deze tekst staat in het kort welke schade niet wordt gedekt. Deze bepalingen staan in het besluit ‘verplichte verzekering bij medisch-wetenschappelijk onderzoek met mensen’. Dit besluit staat op www.ccmo.nl, de website van de Centrale Commissie Mensgebonden Onderzoek (ga naar ‘Bibliotheek’ en dan ‘Wet- en regelgeving’).

De verzekeraar van het onderzoek is:
Naam: CNA Insurance Company Ltd
Adres: Strawinskylaan 703, 1077 XX Amsterdam
Telefoonnummer: 020 57 372 74
Polisnummer: 10201366
Contactpersoon: Mw. Esther van Herk

De verzekering biedt een dekking van € 650.000 per deelnemer, maximaal € 5.000.000 voor het hele onderzoek en maximaal € 7.500.000 zijn per jaar voor alle onderzoeken van dezelfde opdrachtgever.

De verzekering dekt de volgende schade **niet**:

- schade door een risico waarover u in de schriftelijke informatie bent ingelicht. Dit geldt niet als het risico zich ernstiger voordoet dan was voorzien of als het risico heel onwaarschijnlijk was;
- schade aan uw gezondheid die ook zou zijn ontstaan als u niet aan het onderzoek had meegedaan;
- schade door het niet (volledig) opvolgen van aanwijzingen of instructies;
- schade aan uw nakomelingen, als gevolg van een negatief effect van het onderzoek op u of uw nakomelingen;
- schade door een bestaande behandelmethode bij onderzoek naar bestaande behandelmethoden.

Bijlage C: Schema onderzoekshandelingen

	Geschiktheids-bezoek	Maand 0 Start	Maand 1	Maand 2	Maand 3	Maand 6	Maand 9	Maand 12	Extra bezoek *z.n.*
Medische geschiedenis	X								
Reuma medicatie	X	X	X	X	X	X	X	X	X
Geslacht, leeftijd	X								
Roken, alcoholgebruik		X			X	X	X	X	
Lichamelijk onderzoek	X								*z.n.*
Bloeddruk, hartslag		X			X	X	X	X	X
Gewrichts-onderzoek	X	X	X	X	X	X	X	X	X
Biological screening: bloedtest voor hepatitis B en C en tuberculose, zwangerschapstest en röntgenfoto van de longen	X								
Bloedonderzoek		X	X	X	X	X	X	X	X
Röntgenfoto's van handen en voeten		X						X	
Botdichtheids-meting		X						X	
Vragenlijsten		X			X	X	X	X	
z.n. = zo nodig									

Van bovenste onderzoekshandelingen zullen sommigen ook gedaan worden als u niet mee doet aan dit onderzoek, aangezien ze deel zijn van de reguliere zorg voor reumapatiënten. De volgende van de bovenstaande onderzoekshandelingen worden extra gedaan, speciaal voor dit onderzoek:

- Biological screening is extra voor de patienten die behandeld worden met prednison
- Zwangerschapstest (alleen voor vrouwen in de vruchtbare levensfase)
- De röntgenfoto's van handen en voeten
- De botdichtheidsmeting
- De vragenlijsten
- In kaart brengen van medische geschiedenis, voormalige reumamedicatie en roken/alcoholgebruik.

Bijlage D: Toestemmingsformulier deelnemer

Deelname aan de TOPIRA studie

- Ik heb de informatiebrief gelezen. Ook kon ik vragen stellen. Mijn vragen zijn voldoende beantwoord. Ik had genoeg tijd om te beslissen of ik meedoe.

- Ik weet dat meedoen vrijwillig is. Ook weet ik dat ik op ieder moment kan beslissen om toch niet mee te doen of te stoppen met het onderzoek. Daarvoor hoef ik geen reden te geven.

- Ik geef toestemming voor het informeren van mijn huisarts dat ik meedoe aan dit onderzoek.

- Ik geef toestemming voor het informeren van mijn apotheek over de medicatie die ik gebruik.

- Ik geef toestemming voor het verzamelen, gebruiken van mijn gegevens en lichaamsmateriaal voor de beantwoording van de onderzoeksvraag in dit onderzoek, zoals beschreven in de informatiebrief.

- Ik weet dat voor de controle van het onderzoek sommige mensen toegang tot al mijn gegevens kunnen krijgen. Die mensen staan vermeld in deze informatiebrief. Ik geef toestemming voor die inzage door deze personen.

- Ik geef toestemming voor het informeren van mijn huisarts/reumatoloog van onverwachte bevindingen die van belang (kunnen) zijn voor mijn gezondheid.

- Ik weet dat ik niet zwanger mag worden of borstvoeding mag geven tijdens dit onderzoek, omdat de botdichtheidsmetingen en behandeling met tocilizumab in dat geval schadelijk kunnen zijn. Voor patiënten behandeld met tocilizumab geldt dit tot minstens 3 maanden na staken van behandeling met tocilizumab.

- Ik geef toestemming voor het 15 jaar bewaren van mijn persoonsgegevens.

- Ik geef

  □ **wel** □ **geen**

  toestemming om vragenlijsten zoals beschreven in deze informatiebrief naar mijn emailadres te versturen.
- Ik geef

  □ **wel** □ **geen**

  toestemming om mijn persoonsgegevens na dit onderzoek te gebruiken voor toekomstig onderzoek op het gebied van reumatoïde artritis en/of de onderzochte behandelmethode.

- Ik geef

  □ **wel** □ **geen**

  toestemming om mij na dit onderzoek opnieuw te benaderen voor een vervolgonderzoek.

- Ik wil meedoen aan dit onderzoek.

Naam proefpersoon:
Handtekening: Datum : __ / __ / ____

In te vullen door de onderzoeker of diens vertegenwoordiger:
Ik verklaar dat ik deze proefpersoon volledig heb geïnformeerd over het genoemde onderzoek. Als er tijdens het onderzoek informatie bekend wordt die de toestemming van de proefpersoon zou kunnen beïnvloeden, dan breng ik hem/haar daarvan tijdig op de hoogte.

Naam onderzoeker (of diens vertegenwoordiger):
Handtekening: Datum: __ / __ / ____

De proefpersoon krijgt een volledige informatiebrief mee, samen met een getekende versie van het toestemmingsformulier.

Bijlage E: Brochure ‘Medisch-wetenschappelijk onderzoek. Algemene informatie voor de proefpersoon’
Apart als PDF

Bijlage F: Informatiefolder Prednison
Apart als PDF

Bijlage G: Informatiefolder Tocilizumab
Apart als PDF

APPENDIX: monitoring plan (in Dutch)

**1. ALGEMENE GEGEVENS**	
Titel studie	TOPIRA
METC-nummer	Volgt
ABR-nummer	68492.041.19
Deelnemende centra + hoofdonderzoeker	UMCU (prof. J.M. van Laar), Meander Medische Centrum (dr. S.P. Linn-Rasker). *Will be supplemented with additional centers.*
(Centrale) hoofdonderzoeker	Prof. J.M. van Laar
Verrichter	UMC Utrecht
Risicoclassificatie	Verwaarloosbaar = minimale monitoring
Aantal patiënten + looptijd onderzoek	120 patiënten, 2 jaar inclusietijd, 1 jaar follow-up.
Monitor	Julius Center
**2. MONITORING: FREQUENTIE**	
Initiatie visite	Per centrum 1x
Monitor visite	Per centrum: 1 visite per jaar per centrum.
Close-out visite	Per centrum 1 x
**3. MONITORING: INHOUD**	
Inclusiestroom	Controle van de inclusiesnelheid en het uitvalspercentage.
Study File	Per centrum controle aanwezigheid en volledigheid van de betreffende Investigator Site File en papieren Study Files.<br>Bij de verrichter de Study Master File, Center Master File (Beveiligde map op UMC schijf en Castor database).
Informed Consent	Aanwezigheid: minimaal 25% (indien mogelijk 100%) van het totaal aantal geïncludeerde onderzoeksdeelnemers percentrum, indien mogelijk.<br>Navragen van het proces en verificatie op uitvoer van het volledige IC proces bij minimaal 20% van het totaal aantal geïncludeerde onderzoeksdeelnemers percentrum, indien mogelijk.
In- en exclusie criteria	Verificatie van minimaal 20% van het totaal aantal geïncludeerde onderzoeksdeelnemers per centrum, indien mogelijk.
Serious Adverse Events (SAEs/SADEs/SUSARs/USADEs)	De onderzoeksdeelnemers bij wie de steekproef voor de SDV/SDR wordt uitgevoerd worden ook gecontroleerd op niet gerapporteerde SAEs. Daarnaast controle van 20% van de gerapporteerde SAEs/SADEs/SUSARs/USADEs.
Source Data Review en Source Data Verification	Verificatie van minimaal 20% van het totaal aantal geïncludeerde onderzoeksdeelnemers per centrum (SDV en SDR), indien mogelijk.
Onderzoeksproduct	Controleer product accountability van onderzoeksdeelnemers die geselecteerd zijn voor de SDV en welke instructies onderzoeksdeelnemers mee krijgen.
Onderzoeksprocedures	Controleer of instructies voor uitvoer van onderzoeksprocedures

	aanwezig zijn en of onderzoekspersoneel getraind is in het uitvoeren van onderzoeksprocedures.
Onderzoeksdata	Controleer of onderzoeksdata verzameld wordt in een gevalideerde<br>database.
Apparatuur	n.v.t.
Lab & Apotheek	Er wordt gekeken of de apotheek GMP gecertificeerd is.
Aanvullende opmerkingen	Klik hierals u tekst wilt invoeren.

4. MONITORING: VERSLAGLEGGING

De monitor zal een schriftelijk verslag (rapport) maken na ieder bezoek aan een onderzoekslocatie (centrum). Het monitorvisite rapport bevat:

- Een samenvatting van hetgeen de monitor heeft beoordeeld
- Een algemene beschrijving van de kwaliteit
- Een opsomming van belangrijke bevindingen/feiten, afwijkingen en tekortkomingen
- Een overzicht met te nemen maatregelen en aanbevelingen om naleving van het onderzoeksprotocol te garanderen
- De “overall” conclusie

De lokale hoofdonderzoeker ontvangt na ieder bezoek aan het eigen centrum de begeleidende brief en de ‘Monitoring Issues and Actions Tracker’ en dient deze te bewaren in de Investigator Site File. De verrichter*, indien het UMC Utrecht, ontvangt na ieder bezoek aan het eigen of een deelnemend centrum het originele rapport en een origineel (van eigen centrum) of kopie (van deelnemend centrum) van de begeleidende brief en de ‘Monitoring Issues and Action Tracker’. Deze dienen bewaard te worden in de Monocenter Study File (bij monocenter onderzoek), Investigator Site File of Center Master File.

Deze verslaglegging moet door de centrale hoofdonderzoeker en de lokale hoofdonderzoeker bewaard worden en bij een audit ter inzage beschikbaar zijn.
De centrale hoofonderzoeker van de verrichter* (origineel) en de onderzoekslocatie (kopie) ontvangen ook het initiatie visite rapport en het close-out visite rapport. Indien van toepassing zullen ook overige relevante contacten betreffende het onderzoek schriftelijk vastgelegd worden.
Van alle rapportages zal een kopie naar de kwaliteitscoördinator van de divisie waar het onderzoek wordt opgezet/uitgevoerd worden gestuurd.

* Bij onderzoek met een externe verrichter ontvangt de centrale hoofdonderzoeker kopieën in plaats van originele documenten. De lokale hoofdonderzoeker in het UMC Utrecht ontvangt dan de originele documenten.
