# Supplementary material for: Effectiveness of TOcilizumab in comparison to Prednisone In Rheumatoid Arthritis patients with insufficient response to disease-modifying antirheumatic drugs (TOPIRA): study protocol for a pragmatic trial
Source: Trials. 2020 Apr 5;21:313. doi: 10.1186/s13063-020-04260-y (PMC7133012; doi:10.1186/s13063-020-04260-y)
Supplement: Supplementary file 4 — Additional file 4. EULAR and ACR response criteria. [file 13063_2020_4260_MOESM4_ESM.docx]

## Additional file 4: EULAR and ACR response criteria

### The EULAR response criteria are defined as follows:

| Improvement  in DAS28  Present DAS28 | > 1.2 | > 0.6 and ≤ 1.2 | ≤ 0.6 |
| --- | --- | --- | --- |
| ≤ 3.2 | Good response | Moderate response | No response |
| > 3.2 and ≤ 5.1 | Moderate response | Moderate response | No response |
| > 5.1 | Moderate response | No response | no response |

### ACR response criteria are defined as follows:

- ACR20 is ≥ 20% improvement
- ACR50 is ≥ 50% improvement
- ACR70 is ≥ 70% improvement

Improvement is defined as ≥ 20%, ≥ 50% or ≥ 70% improvement in tender AND swollen joint count AND in ≥ 3 of the following parameters:

1. Patient assessment of pain
2. Patient assessment of global disease activity
3. Physician assessment of global disease activity
4. Patient assessment of physical function, e.g. using the health assessment questionnaire
5. Acute phase reactants (ESR or CRP)
